# Genomic Analysis of Bacteriophage BUCT86 Infecting Klebsiella Pneumoniae

**DOI:** 10.1128/mra.01238-21

**Published:** 2022-04-11

**Authors:** Ke Han, Yinuo Zhu, Fei Li, Mengzhe Li, Xiaoping An, Lihua Song, Huahao Fan, Yigang Tong

**Affiliations:** a College of Life Science and Technology, Beijing University of Chemical Technology, Beijing, China; b Beijing Advanced Innovation Center for Soft Matter Science and Engineering, Beijing University of Chemical Technology, Beijing, China; Loyola University Chicago

## Abstract

Phage BUCT86 possesses a genome of 44,542 bp of double-stranded DNA, with a G+C content of 54%. The result of BLASTn analysis showed that the genome sequence of phage BUCT86 shared similarity with that of Klebsiella phage CX1, with 82% query coverage and 93.31% identity.

## ANNOUNCEMENT

Klebsiella pneumoniae is a common Gram-negative opportunistic pathogen that causes a variety of infectious diseases, including urinary tract infections, bacteremia, pneumonia, and liver abscesses (1). With the wide application of antibiotics, K. pneumoniae has shown obvious drug resistance (2). Meanwhile, the emergence of highly virulent strains has increased the difficulty of infection control. In order to alleviate the spread of bacterial resistance, it is urgent to develop antibacterial agents that can supplement or replace antibiotics. Bacteriophages (phages), viruses that infect bacteria, have attracted much attention because of their potential to treat drug-resistant bacterial infections and have been successfully applied around the world (3).

**FIG 1:**
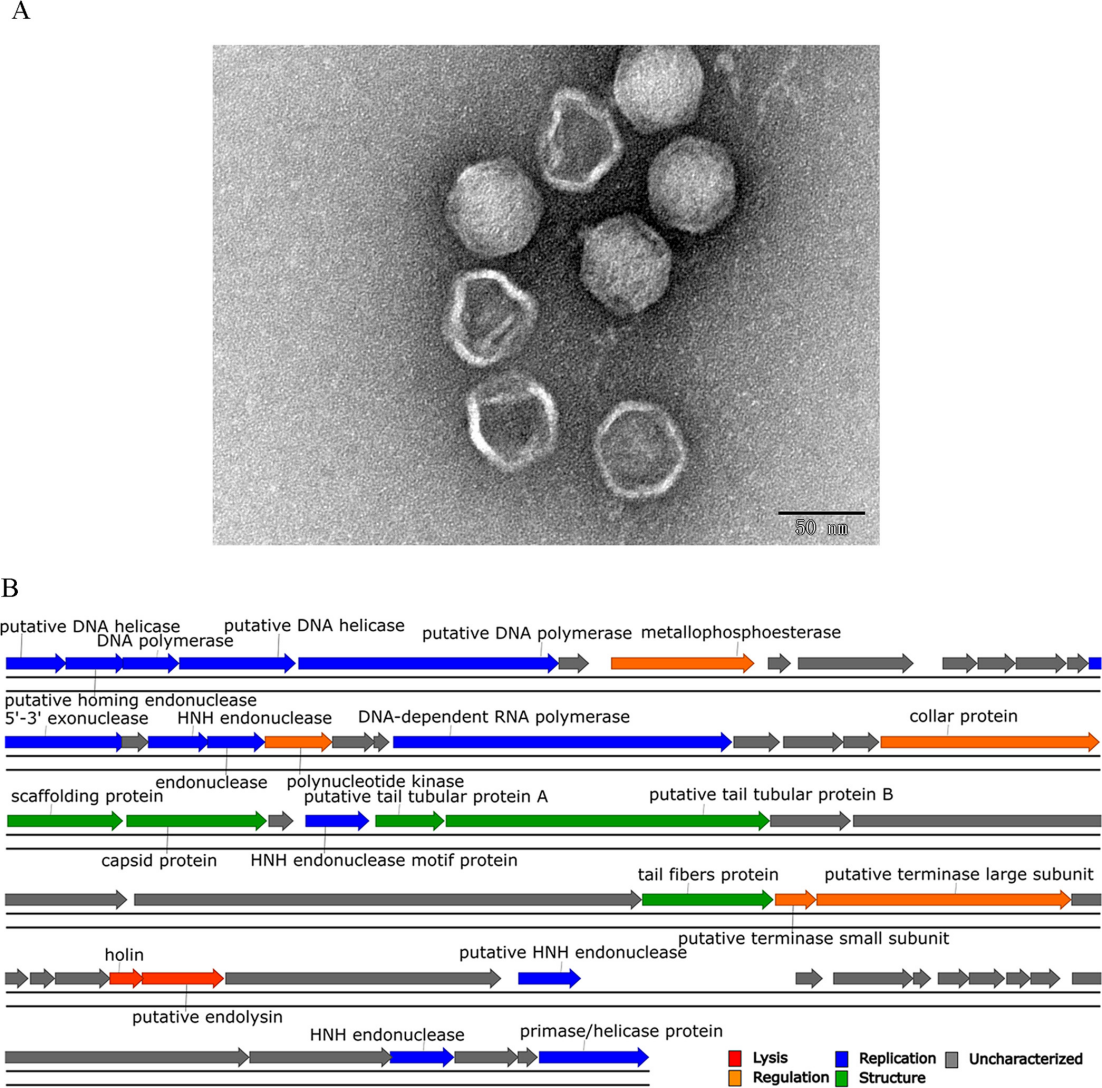
TEM and genome function map of phage BUCT86. (A) TEM of phage BUCT86. Scale bar, 50 nm. Four virions were measured in the figure. (B) Genome function map of phage BUCT86. Different colors refer to different functional categories.

In this study, phage BUCT86 was isolated from hospital sewage (Beijing, China) using K. pneumoniae strain 2773 as the host. Briefly, hospital sewage was centrifuged, filtered through a 0.22-mm filter, and then used directly for spot testing to verify the presence of phage. The purification of phage was performed three times by taking a single plaque with the double-layer agar method (4). The phage crude extract was concentrated by ultracentrifugation, and highly purified virus particles were obtained by sucrose density gradient centrifugation (5). The phage particles were observed with a JEM-1200EX transmission electron microscope (JEOL Ltd., Tokyo, Japan) at an accelerating voltage of 80 kV. Transmission electron microscopy (TEM) showed that the head diameter of the phage was 53.88 ± 1.82 nm and the tail length was 5.86 ± 0.17 nm. Based on the morphological characteristics, phage BUCT86 belongs to the family *Podoviridae* (Fig. 1A).

The genomic DNA of phage BUCT86 was extracted using a DNA extraction kit (Omega Bio-Tek, Norcross, GA, USA). The phage sequencing library was prepared with the New England BioLabs NEBNext Ultra II kit v3 and sequenced using Illumina HiSeq 2500 paired-end sequencing technology, with an average read length of 150 bp; 1,124,079 filtered paired-end reads were obtained by Trimmomatic v0.36, with a coverage depth of about 20.38×. Finally, contigs were assembled by SPAdes v3.13.0 (6). The genome functional map was prepared using the CLC Genomics Workbench v9 and optimized using Inkscape v0.92.1. All tools were run with default parameters unless otherwise noted.

Phage BUCT86 has a linear double-stranded DNA genome consisting of 44,542 bp, with a G+C content of 54%. PhageTerm showed that the ends of the sequence were random. The genome characteristics are shown in Fig. 1B. Through BLASTn comparison (https://blast.ncbi.nlm.nih.gov/Blast.cgi), we found that phage BUCT86 was similar to Klebsiella phage CX1 (GenBank accession number MT090077), with 82% query coverage and 93.31% identity.

The genome sequences were annotated by RAST (https://rast.nmpdr.org) (7) and then checked by BLASTp with the NCBI database (https://www.ncbi.nlm.nih.gov) (8). Phage BUCT86 had 57 open reading frames (ORFs), of which only 25 had predicted functions; the rest were annotated as hypothetical proteins (Table 1).

**TABLE 1:** Predicted ORFs in the genome of phage BUCT86

ORF	Nucleotide position		Strand^*a*^	Predicted function	Best match	GenBank accession no. for best match	E value	Coverage (%)	Identity (%)
	Start	Stop							
ORF1	1	441	F	Putative DNA helicase	Klebsiella phage vB_KpnP_fHeKpn01	QFG06564.1	2.00E−101	100	99.32
ORF2	434	880	F	Putative homing endonuclease	Klebsiella phage vB_KpnP_SU552A	CAD5239093.1	1.00E−49	90	65.71
ORF3	849	1262	F	DNA polymerase	Klebsiella phage F19	YP_009006036.1	1.00E−91	100	100
ORF4	1262	2110	F	Putative DNA helicase	Klebsiella phage vB_KpnP_KpV48	YP_009787571.1	0	96	99.63
ORF5	2130	4028	F	Putative DNA polymerase	Klebsiella phage VLC6	QJI52592.1	0	100	98.26
ORF6	4025	4246	F	Hypothetical protein	Escherichia phage vB_EcoP_ZX6	QXO10409.1	2.00E−43	100	98.63
ORF7	4408	5451	F	Metallophosphoesterase	Klebsiella phage vB_KpnP_KpV74	YP_009789259.1	0	100	99.14
ORF8	5550	5717	F	Hypothetical protein	Klebsiella phage phiKpS2	YP_009792375.1	8.00E−27	100	98.18
ORF9	5769	6611	F	Hypothetical protein	Klebsiella phage Pone	QPB09063.1	0	100	97.50
ORF10	6822	7076	F	Hypothetical protein	Klebsiella phage KMI6	QEG10143.1	2.00E−45	100	92.86
ORF11	7077	7355	F	Hypothetical protein	Klebsiella phage 1 TK-2018	AZF88729.1	3.00E−48	98	85.71
ORF12	7355	7726	F	Hypothetical protein	Klebsiella phage vB_KpnP_SU552A	YP_009204814.1	2.00E−76	100	95.93
ORF13	7729	7887	F	Hypothetical protein	Klebsiella phage vB_KpnP_SU552A	YP_009204817.1	1.00E−27	100	98.08
ORF14	7887	8855	F	5′-3′ exonuclease	Klebsiella phage phiKpS2	YP_009792381.1	0	100	100
ORF15	8812	9012	F	Hypothetical protein	Klebsiella phage KpV475	YP_009280697.1	4.00E−39	100	100
ORF16	9006	9455	F	HNH endonuclease	Klebsiella phage VLC1	QGZ00740.1	6.00E−99	100	90.60
ORF17	9437	9859	F	Endonuclease	Klebsiella phage VLC1	QGZ00741.1	3.00E−97	100	99.29
ORF18	9856	10350	F	Polynucleotide kinase	Klebsiella phage MEW1	QOQ37707.1	1.00E−107	99	92.64
ORF19	10347	10661	F	Hypothetical protein	Klebsiella phage vB_KpnP_SU552A	YP_009204822.1	1.00E−69	100	100
ORF20	10648	10764	F	Hypothetical protein	Klebsiella phage vB_KpnP_KpV48	YP_009787591.1	6.00E−19	100	94.74
ORF21	10792	13260	F	DNA-dependent RNA polymerase	Klebsiella phage KP34	YP_003347629.1	0	100	98.18
ORF22	13270	13608	F	Hypothetical protein	Klebsiella phage vB_KpnP_KpV74	YP_009789274.1	3.00E−75	100	98.21
ORF23	13632	14072	F	Hypothetical protein	Klebsiella phage SRD2021	QWY13527.1	5.00E−102	100	98.63
ORF24	14069	14332	F	Hypothetical protein	Klebsiella phage KP34	YP_003347631.1	8.00E−51	100	100
ORF25	14342	15937	F	Collar protein	Klebsiella phage Kp2	YP_009188350.1	0	100	99.25
ORF26	15952	16794	F	Scaffolding protein	Klebsiella phage AltoGao	YP_009792083.1	0	100	98.57
ORF27	16820	17842	F	Capsid protein	Klebsiella phage KP-Rio/2015	YP_009787381.1	0	99	98.53
ORF28	17854	18036	F	Hypothetical protein	Klebsiella phage KP34	YP_003347637.1	8.00E−31	100	98.33
ORF29	18124	18588	F	HNH endonuclease motif protein	Klebsiella phage SRD2021	QWY13533.1	2.00E−105	100	97.40
ORF30	18633	19136	F	Putative tail tubular protein A	Klebsiella phage vB_KpnP_KpV48	YP_009787600.1	8.00E−118	100	98.20
ORF31	19146	21506	F	Putative tail tubular protein B	Klebsiella phage vB_KpnP_SU552A	YP_009204831.1	0	100	98.09
ORF32	21508	22095	F	Internal virion protein	Klebsiella phage NTUH-K2044-K1-1	YP_009098376.1	2.00E−137	100	98.97
ORF33	22112	24796	F	Internal virion protein	Klebsiella phage CX1	QIN95022.1	0	100	99.11
ORF34	24847	28545	F	Putative internal core protein	Klebsiella phage Kp_Pokalde_001	QWT56635.1	0	100	98.94
ORF35	28547	29503	F	Tail fiber protein	Klebsiella phage phiBO1E	YP_009784844.1	0	100	93.40
ORF36	29515	29817	F	Putative terminase small subunit	Klebsiella phage AltoGao	YP_009792092.1	9.00E−61	100	97.00
ORF37	29817	31673	F	Terminase large subunit	Klebsiella phage vB_KpnP_SU503	YP_009199931.1	0	100	99.68
ORF38	31673	32047	F	Hypothetical protein	Klebsiella phage vB_KpnP_Bp5	QDJ96108.1	1.00E−81	100	99.19
ORF39	32059	32241	F	Hypothetical protein	Klebsiella phage vB_KpnP_KpV48	YP_009787609.1	9.00E−32	100	100
ORF40	32241	32645	F	Hypothetical protein	Klebsiella phage KpV41	YP_009188794.1	9.00E−87	100	99.25
ORF41	32638	32889	F	Holin	Klebsiella phage phiKpS2	YP_009792406.1	3.00E−51	100	97.59
ORF42	32873	33472	F	Putative endolysin	Klebsiella phage KMI6	QEG10116.1	3.00E−140	100	96.48
ORF43	33482	35491	F	Hypothetical protein	Klebsiella phage vB_KpnP_KpV48	YP_009787613.1	0	100	98.21
ORF44	35616	36071	F	Putative HNH endonuclease	Klebsiella phage KpV71	YP_009302757.1	1.00E−99	98	91.28
ORF45	37636	37833	F	Hypothetical protein	Klebsiella phage vB_KpnP_IME337	QEQ50440.1	4.00E−37	100	95.38
ORF46	37910	38494	F	Hypothetical protein	Klebsiella phage vB_KpnP_fHeKpn01	QFG06550.1	5.00E−135	100	97.94
ORF47	38491	38622	F	Hypothetical protein	Klebsiella phage CX1	QIN95036.1	5.00E−21	100	93.02
ORF48	38672	38905	F	Hypothetical protein	Klebsiella phage VLC5	QIW86374.1	1.00E−47	100	94.81
ORF49	38898	39161	F	Hypothetical protein	Klebsiella phage vB_KpnP_SU552A	YP_009204794.1	1.00E−54	100	96.55
ORF50	39170	39349	F	Hypothetical protein	Klebsiella phage phiKpS2	YP_009792361.1	1.00E−34	100	98.31
ORF51	39346	39564	F	Hypothetical protein	Klebsiella phage Pone	QPB09047.1	9.00E−46	100	97.22
ORF52	39649	41628	F	Hypothetical protein	Klebsiella phage vB_KpnP_KpV74	YP_009789248.1	0	100	89.55
ORF53	41628	42671	F	Putative peptidase	Klebsiella phage F19	YP_009006030.1	0	100	95.39
ORF54	42652	43119	F	HNH endonuclease	Klebsiella phage Pone	QPB09055.1	2E−30	93	41.38
ORF55	43122	43589	F	Hypothetical protein	Klebsiella phage vB_KpnP_SU552A	YP_009204800.1	1.00E−93	98	88.89
ORF56	43582	43728	F	Hypothetical protein	Klebsiella phage VLC5	QIW86382.1	7.00E−16	97	78.72
ORF57	43738	44538	F	Primase/helicase protein	Escherichia phage vB_EcoP_ZX6	QXO10414.1	0	99	97.74

a F, forward.

Genomic analysis of Klebsiella phage BUCT86 may provide insights for the development of phage therapy against Klebsiella, as well as enriching the knowledge about the diversity of Klebsiella phages.

### Data availability.

The complete genome sequence of phage BUCT86, with annotations, was submitted to the GenBank database under the accession number OL474125. The raw sequence reads were deposited in the NCBI SRA under the accession number SRR17406163.
